# Simulations of symptomatic treatments for Alzheimer's disease: computational analysis of pathology and mechanisms of drug action

**DOI:** 10.1186/alzrt153

**Published:** 2012-11-26

**Authors:** Patrick D Roberts, Athan Spiros, Hugo Geerts

**Affiliations:** 1 Department of Biomedical Engineering, Oregon Health & Science University, 3303 SW Bond Avenue, Portland, OR 97239 USA; 2 In Silico Biosciences, Inc., 405 Waltham Street, Lexington, MA 02421 USA

## Abstract

**Introduction:**

A substantial number of therapeutic drugs for Alzheimer's disease (AD) have failed in late-stage trials, highlighting the translational disconnect with pathology-based animal models.

**Methods:**

To bridge the gap between preclinical animal models and clinical outcomes, we implemented a conductance-based computational model of cortical circuitry to simulate working memory as a measure for cognitive function. The model was initially calibrated using preclinical data on receptor pharmacology of catecholamine and cholinergic neurotransmitters. The pathology of AD was subsequently implemented as synaptic and neuronal loss and a decrease in cholinergic tone. The model was further calibrated with clinical Alzheimer's Disease Assessment Scale-cognitive subscale (ADAS-Cog) results on acetylcholinesterase inhibitors and 5-HT6 antagonists to improve the model's prediction of clinical outcomes.

**Results:**

As an independent validation, we reproduced clinical data for apolipoprotein E (APOE) genotypes showing that the ApoE4 genotype reduces the network performance much more in mild cognitive impairment conditions than at later stages of AD pathology. We then demonstrated the differential effect of memantine, an N-Methyl-D-aspartic acid (NMDA) subunit selective weak inhibitor, in early and late AD pathology, and show that inhibition of the NMDA receptor NR2C/NR2D subunits located on inhibitory interneurons compensates for the greater excitatory decline observed with pathology.

**Conclusions:**

This quantitative systems pharmacology approach is shown to be complementary to traditional animal models, with the potential to assess potential off-target effects, the consequences of pharmacologically active human metabolites, the effect of comedications, and the impact of a small number of well described genotypes.

## Introduction

As diseases progress, different treatment strategies may be necessary to compensate for changing bio-logical conditions. Therefore, we need to estimate how and when such changes take place so that the treatment may be altered in pace as the disease progresses. However, unless specific biomarkers are available to directly measure progression of the disease, we must rely on indirect functional indicators to signal the progress. For complex diseases such as Alzheimer's disease (AD), biophysical modeling can provide an important tool [1] to link indirect functional indicators with the underlying biological process and predict both the timing and mechanisms that indicate effective treatments at various stages of the disease.

Many experimental therapeutics in AD are based on disease-modifying strategies, yet the ultimate clinical test is functional. Although cognitive outcome is dependent upon integrity of the underlying neuronal structures, cognition is modulated by the interaction of many neuromodulatory systems that have been primary targets of medications. The only approved medications for AD are based on the cholinergic system [2], and specific muscarinic [3] and nicotinic targets [4] are currently under investigation. Other symptomatic interventions under investigation include serotonergic targets, such as 5-HT_4_[5] and a 5-HT_6_[6,7]. However, these treatments are most effective during the middle stages of the disease, after mild cognitive impairment (MCI) develops into AD, and before the late stages.

**Figure 1:**
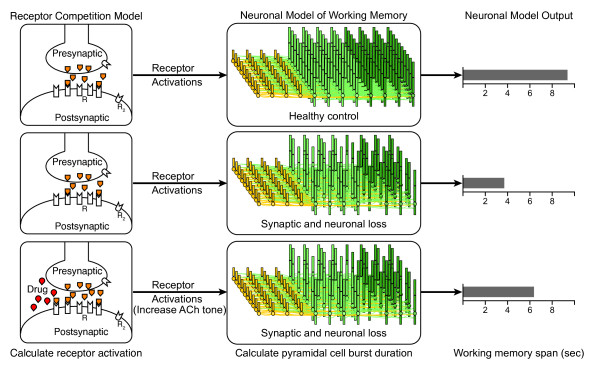
**Overview of modeling platform**. The modeling platform consists of two components, a receptor competition model (left column) and a biophysical neuronal network model (center column). The receptor competition model calculates the activation of receptors for neuromodulators (M1, α7, α4β2 receptors for acetylcholine; D1, D4 receptors for dopamine; 5-HT1A, 5-HT2A, 5-HT3 receptors for serotonin; α2A receptors for norepinephrine) for the region of the brain represented by the neuronal network model. The receptor activations are used to adjust modulate synaptic and membrane currents in the network model. The output of the network model simulates a burst of pyramidal cell activity as a measure of working memory (right column). The platform is initially calibrated to generate an average working memory span of over 9 sec (top row), and a disease pathology such as loss of neurons and synapses reduces the working memory span (middle row). Changes in the receptor activations, such as increased cholinergic tone change the working memory span (bottom row) to reduce the symptoms.

In order to provide better guidance on clinical candidate development, we have developed a conductance-based, biophysical model of cortical networks to simulate the progression of AD. The model represents disease pathologies as neuronal and synaptic loss and changes in cholinergic tone. Neuromodulatory effects are included by calculating receptor activations in the presence of normal and pathological levels of modulators and drugs, and then coupling receptor activation to biophysical changes in the network. To link these pathologies to cognitive function, we simulate a working memory task and calibrate the outcome with clinical data (Figure 1).

The calculated measure of working memory is modified by pathology such as synaptic loss and by changes in the receptor activations. This output of the model, the working memory span, is used to calibrate the receptor parameters with a clinical database. The calibrated model represents the underlying state of the cortex during each stage of the disease, and predicts the systems level changes caused by interventions that lead to changes in functional symptoms. Predictions using the calibrated model include the transition from MCI to AD, and the progression of pathology in synaptic and neuronal loss throughout the disease.

We also demonstrate the mechanism of action of memantine, an N-Methyl-D-aspartic acid (NMDA) receptor inhibitor, on late stage AD. We show that the loss of excitatory neurons in late stage AD shifts the excitatory-inhibitory balance in cortical circuitry so that memantine improves cognitive function. Memantine is currently approved for the treatment of moderate-to-severe AD and has shown clinical benefit in these patients [8], but the mechanism of action has not previously been clearly demonstrated.

These results suggest that computational-based modeling [9] could become a critical tool for improving pharmaceutical research and development, especially for complex diseases [1].

## Methods

The computational model has two main components, a receptor competition model [10] and a conductance-based cortical model [11] as shown in Figure 1. The receptor competition model is used to calculate the activation of modulator receptors that influence the neurons and synaptic conductances in the cortical model. The cortical model simulates the activity of cortical pyramidal cells and inhibitory interneurons to estimate the burst firing duration associated with a working memory task. The spiking activity of the cortical model is compared with clinical data to calibrate the model and analyze the progression of AD and mechanisms of action for symptomatic treatments.

### The receptor competition model

We implemented a receptor competition model to simulate the competition between neurotransmitter, drug, its metabolite and a possible radiotracer [10]. We use this model to calculate the postsynaptic serotonergic and cholinergic receptor activation for different clinical conditions because 5-HT_6 _antagonists have been tested in different doses and acetylcholinesterase inhibitors (AChE-I) increase free ACh at different doses. This receptor competition model is a set of ordinary differential equations that describes the time-dependent changes in pre- and postsynaptic receptor activations, neurotransmitter and drug levels in the synaptic cleft and amount of binding to different receptors.

If [*NT*] is the free neurotransmitter (for instance 5-HT) concentration and [*R_f_*] is the concentration of free receptors, then the change in receptors bound by neurotransmitter, [*R_n_*], is governed by a system of four ordinary differential equations [12],

(1) $$\frac{d\left[R_{i}\right]}{dt}=k_{on}^{i}\cdot \left[NT\right]\cdot \left[R_{f}\right]-k_{on}^{i}\cdot K_{d}^{i}\cdot \left[R_{i}\right]$$

where the super(sub)script *i *has four possible values (*n *= neurotransmitter, *d *= drug, *m *= metabolite and *t *= tracer). Combined with the continuity equation, *R_f _*= *R_o_*− *R_n_*− *R_d_*− *R_m_*− *R_t_*, the system of differential equations is solved numerically to obtain the activation (*R_o _*= concentration of receptors). The initial condition that all receptors begin in the free state (subscript *n *refers to the neurotransmitter), and in general, $K_{d}^{n}=K_{off}^{n}/k_{on}^{n}$. All differential equations are integrated with a fourth-order Runge-Kutta algorithm with a time step of 0.01 msec using proprietary custom software written in Java.

The amount of free neurotransmitter depends on two processes, exponential decay and quantal release. Exponential decay is classically defined as [*NT*] (*t*) = [*NT*(0)] exp(−*t *ln(2)/*τ*_1/2_) where *τ*_1/2 _is the half-life of the decay process. The amount of presynaptic receptor activation which occurred 150 ms before the current release event then determines the amount of new release, *r_new_*, as follows

(2) $$r_{new}=r_{0}\left[1+r_{max}\left(1-2\frac{A^{S}}{A^{S}+{B_{0}}^{S}}\right)\right]$$

where *r*_0 _is the base release amount, *r_max_*is the maximum relative change for release, *A *is the receptor activation at the specified time in the past, *S *is the sensitivity to presynaptic receptor and *B*_0 _is the amount of normal presynaptic binding that one would expect in the tonic case. All differential equations are solved with a fourth-order Runge-Kutta method with a time step of 0.01 msec.

In addition, the release can be modulated by a depression or facilitation mechanism [13]. Instead of using internal Ca^++ ^levels to determine neurotransmitter release, we consider the facilitation and depression based solely on the amount of time elapsed since the previous firing using a phenomenological equation. If we denote the time of the *n*th firing by *t_n_*, then the release amount is modified based on all previous firings as follows

(3) $$r_{f}=r_{0}\left(1+\overset{n-1}{\underset{i=1}{\sum }}w_{f}\text{exp}\left[-k_{f}\left(t_{n}-t_{i}\right)\right]-w_{d}\text{exp}\left[-k_{d}\left(t_{n}-t_{i}\right)\right]\right)$$

where *w_f_*is the facilitation weight, *w_d_*is the depression weight, *k_f_*is the decay rate of facilitation and *k_d_*is the decay rate of depression. The simulation is initiated by first finding the equilibrium given a constant amount of free neurotransmitter at 500 nM and then goes on for a transitory time of 5 seconds at the predetermined tonic firing rate. Finally, the simulation runs for an additional 10 seconds during which time average binding levels are determined. While *k_on_*and *k_off_*parameters are determined experimentally, all the parameters that describe the presynaptic neurotransmitter physiology are calibrated with preclinical experiments using rapid-cyclic fast voltammetry on levels of neurotransmitters.

Using the competition model between neurotransmitter, drug and tracer for binding at the postsynaptic receptor, we determined the drug concentration that corresponds to a clinically measured radiotracer displacement. This value for the drug concentration is the free and functional intra-synaptic concentration that is dependent upon the pharmacokinetic properties of the drug and was used in further calculations.

*The Cholinergic synapse model*. As the mainstay of Alzheimer therapy are cholinomimetic drugs such as Acetylcholinesterase inhibitor (AChE-I), it is necessary to have a well calibrated computer model of the cholinergic synapse [14]. Briefly, the presynaptic autoregulation of cholinergic neurotransmission is regulated by M_2 _muscarinic receptor (mACh-R) [15], the physiology of which has been studied using M_2 _receptor knockout mice [16]. Results on the pharmacological effects of oxotremorine and muscarine on quantal ACh release in wild-type and M_2 _receptor knockout provide biological data for which the negative autoreceptor coupling parameters were calibrated. Presynaptic release of endogenous ACh is further synchronized with firing frequencies of the cholinergic nerve endings, which are typically in the 6-8 Hz range [17,18].

Removal of ACh from the cholinergic cleft is mediated by the activity of the acetylcholinesterase enzyme, one of the fastest enzymes in the human body. The pEC50 for ACh hydrolysis by AChE is -6.6 with a hill slope of 0.9, while the enzyme saturates at a maximal turnover rate of 25,000/sec [19]. The density of AChE molecules is 2400/sq-micron [20] for the neuromuscular junction. This constrains the values for the half-life of the free ACh.

The amount of ACh released per action potential is in the range of 7,000 to 10,000 molecules as measured by fluctuation analysis [21], although more recent data in frog muscle acetylcholine receptors suggest single quanta of 70 molecules and the release of 5-6 quanta per action potential [22]. The volume of a cholinergic synapse can be estimated from EM studies; for instance the cholinergic synapse in the ventral tegmental area (VTA) can cover an area of 1 μm^2^[23]. Assuming a distance between pre-and postsynaptic membrane ranging between 0.05 and 0.2 μm, our estimate for the volume of a cholinergic synapse ranges from 5-20×10^-17 ^l. A concentration of 1uM corresponds to 30 to 120 molecules in the synaptic cleft. This number serves as the basis for the amount of ACh released per action potential and further calibration of the cholinergic synapse [14].

### The cortical network model

We extended a biophysically realistic model of a network comprised of four-compartment pyramidal cells and two-compartment gamma-Aminobutyric acid (GABA) interneurons [11,24] with the receptor physiology of 18 different dopaminergic, serotonergic, noradrenergic, and cholinergic receptors. We describe here only a brief overview of the models to emphasize the changes relative to the published article [11].

For more precise implementation of the Alzheimer's pathology, we extended the network to a system of 80 pyramidal cells and 40 interneurons and we modified the connectivity strength so that it had similar firing frequencies for the different types of neurons as the 30 neuron network [11].

We added the receptor physiology of 18 different dopaminergic, serotonergic, noradrenergic, and cholinergic receptors to the NMDA, α-amino-3-hydroxy-5-methyl-4-isoxazolepropionic acid ApoE4 (AMPA) and GABA-A receptors already present in the published source [11]. The circuit connectivity is based on estimations from the relative number of pyramidal cells and interneurons [24] that synapse onto each other. In our implementation 40% of the interneurons do not synapse with pyramidal cells, but form a small recurrent network between themselves and other inhibitory interneruons. This network has some 50,000 synapses. Basically, the network is initially calibrated using single-cell recordings in primates [11,25].

The receptor couplings are based on documented intracellular processes with these receptors and are calibrated using the correlation between the effect of therapeutic interventions in the network and their clinical working memory performance on the N-Back test in both normal subjects and schizophrenia patients.

An mGluR5-dependent delayed after-depolarization current was added to the model to increase the spiking rate of pyramidal cells for several seconds and was implemented as an α-function in the model with a time constant similar to the observation in [26]. In contrast to the network in [11], 40% of the interneurons do not synapse with pyramidal cells but synapse with other inhibitory interneurons that connect to pyramidal cells to be consistent with the relative number of pyramidal cells and interneurons [24].

**Figure 2:**
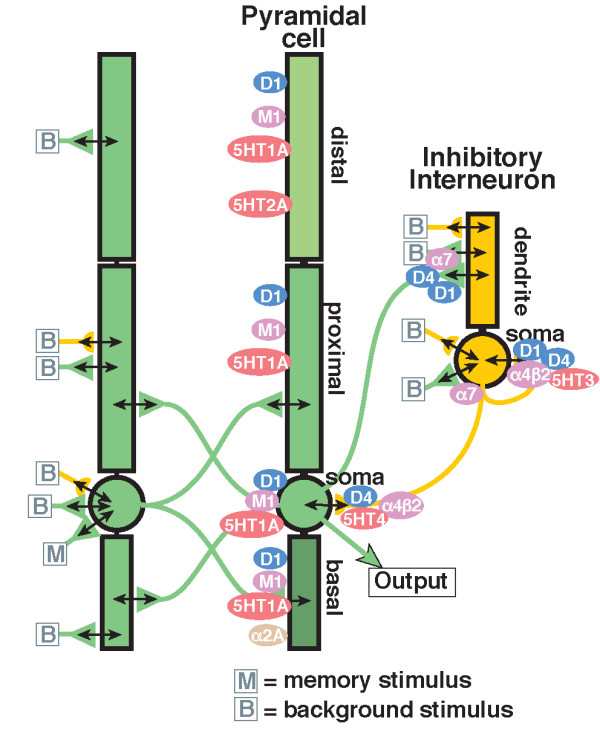
**Schematic diagram of the connectivity and receptors in the prefrontal cortex network**. Schematic diagram of the connectivity and receptors in the prefrontal cortex network. The position of different types of cholinergic (M_1_, *α*_7_, *α*_4_*β*_2_), dopaminergic (D_1_, D_4_), serotonergic (5-HT_1*A*_, 5-HT_2*A*_, 5-HT_3_), noadrenergic (*α*_2*A*_), glutamate and GABA receptors is shown according to their preclinical data. The memory stimulus (M) is given at 2 sec into the simulation and represents a sensory or conceptual stimulus that is introduced into the network. The background noise (B) represents the interaction of this particular network with the rest of the cortex and the brain and is described as a Poisson process and is present throughout the simulation.

Figure 2 shows the general architecture of the cortical network. Each pyramidal cell consists of a distal dendrite, a proximal dendrite, a cell soma and an apical dendrite, whereas an interneuron has only a cell soma and a dendritic compartment. While all pyramidal cells synapse with themselves to form a recurrent network and all of them make synapses with interneurons, only 60% of the interneurons synapse with pyramidal cells; the remaining 40% form an internal recurrent network. There is a constant background synaptic noise [11] that simulates the effect of the neurons that are not represented explicitly in the model. A few receptors that modulate the glutamatergic and GABA-ergic connections are also shown in Figure 2. Modulation of the connections is implemented by scaling the maximum conductance of the synaptic connection proportionally to the activation of each receptor. Excitatory AMPA and NMDA synapses onto pyramidal cells are modulated by D1R with a factor of $\left(1+P_{D1}^{syn}\cdot A_{D1}\right)$, where *A*_*D*1 _is the relative difference of D1R activation in the presence of the drug compared with the control D1R activation and $P_{D1}^{syn}$ is the coupling parameter that is calibrated using clinical data. In addition, excitatory synapses onto pyramidal cells are modulated by M2 receptors via an α7 mechanism that is implemented similarly. Excitatory synapses onto inhibitory interneurons are modulated similarly except that AMPA receptors are modulated by an additional factor due to D4R activation.

Inhibitory GABA-A synapses are also modulated by D1R as with excitatory neurons, but have independent coupling parameters. In addition, GABA-A synapses are modulated by M2 receptors via an *α*_4_*β*_2 _mechanism implemented similarly as for excitatory synapses. Inhibitory GABA-A synapses onto inhibitory interneurons are further modulated by 5-HT_3 _receptors.

Changes in membrane potential were calculated using partial differential equations that were integrated using the simulation package NEURON [27]. The time course of the membrane potential, *V*, was determined by integrating the following equation:

(4) $$C\frac{\partial }{\partial t}\left(V\right)=I_{Kdr}+I_{KCa}+\ldots .$$

where *C *is the membrane capacitance and *I_a _*= *g_a_*(*V *− *E_a_*) is a term in the sum of membrane currents described in detail in [11]. The currents are calculated by the voltage-dependent ionic conductance *g_a_*of an *a*-type ion channel, and *E_a_*is the reversal potential of an *a*-type ion channel.

The voltage-sensitive ionic conductances were calculated using activation (*x_a_*) and inactivation (*y_a_*) variables:

(5) $$g_{a}={ḡ}_{a}x_{a}^{n_{a}}y_{a}^{m_{a}},\;\text{and}\;\frac{dx_{a}}{dt}=\frac{1}{\tau_{x_{a}}}\left(x_{a}^{\infty }-x_{a}\right),$$

where ${ḡ}_{a}$ is the maximum ionic conductance, and *n_a_*is the power of the activation variable. The asymptote $\left(x_{a}^{\infty }\right)$ and decay $\left(\tau_{x_{a}}\right)$ constants are functionally related to the first-order rate constants in the Hodgkin-Huxley formulation [28]. The gating variables are themselves dependent on the membrane potential, *V*, through empirically derived relationships for each channel type so that $x_{a}^{\infty }$ and $\tau_{x_{a}}$ are defined by voltage-dependent gating variables, α(*V*) and *β*(*V*). The inactivation variables obey a similar first-order equation (see Table 1 for parameters and Table 2 for gating functions used in this model).

**Table 1:** Compartment parameters.

Compartment	Length, *μ*m	Diameter, *μ*m	gNaf	gNap	gHva	gKdr	gKs	gCa
Pyr - Apical-distal	400.0	2.6	0.028	0	0.000255	0.0092	0.00018	0.0022

Pyr- Proximal-distal	400.0	2.6	0.028	0.001	0.00063	0.0092	0.00018	0.0038

Pyr - Basal	150.0	16.0	0.028	0.001	0.00063	0.0092	0.00018	0.0038

Pyr - Soma	86.3	6.14	0.086	0.0022	0.000306	0.0338	0.000105	0.0022

Int - Dendrite	150.0	10.0	0.02	-	-	0.008	-	-

Int - Soma	15.0	15.0	0.1	-	-	0.04	-	-

Parameters adapted from [11] for dimensions and current densities of the model compartments.

**Table 2:** Compartment parameters.

Current	*m*	*α*	*β*	*x*_∞_	*τ_x _*(ms)	
Pyr - Naf	3	$\frac{-0.2816\left(V+28\right)}{-1+\text{exp}\left(-\left(V+28\right)/9.3\right)}$	$\frac{0.2464\left(V+1\right)}{-1+\text{exp}\left(\left(V+1\right)/6\right)}$	$\frac{\alpha }{\alpha +\beta }$	$\frac{1}{\alpha +\beta }$	

	1	$\frac{0.098}{\text{exp}((V+43.1)/20}$	$\frac{1.4}{1+\text{exp}\left(-\left(V+13.1\right)/10\right)}$	$\frac{\alpha }{\alpha +\beta }$	$\frac{1}{\alpha +\beta }$	

Pyr - Kdr	4	$\frac{-0.018\left(V-13\right)}{-1+\text{exp}\left(-\left(\left(V-13\right)/25\right)\right)}$	$\frac{0.0054\left(V-23\right)}{-1+\text{exp}\left(\left(V-23\right)/12\right)}$	$\frac{\alpha }{\alpha +\beta }$	$\frac{1}{\alpha +\beta }$	

Pyr - Nap	1	$\frac{-0.2816\left(V+17\right)}{-1+\text{exp}\left(-\left(V+17\right)/9.3\right)}$	$\frac{0.2464\left(V-10\right)}{-1+\text{exp}\left(\left(V-10\right)/6\right)}$	$\frac{\alpha }{\alpha +\beta }$	$\frac{1}{\alpha +\beta }$	

	1	$0.00002\text{exp}\left(-\frac{V+42.8477}{4.0248}\right)$	$\frac{0.014286}{1+\text{exp}\left(-\frac{V-413.9284}{148.2589}\right)}$	$\frac{\alpha }{\alpha +\beta }$	$\frac{1}{\alpha +\beta }$	

Pyr - HVA	2	-	-	$\frac{1}{1+\text{exp}\left(-\frac{V+24.6}{11.3}\right)}$	1.25 sech(0.031(*V *+ 37.1))	

	2	-	-	$\frac{1}{1+\text{exp}\left(-\frac{V+12.6}{18.9}\right)}$	420	

Pyr - Ks	1	-	-	$\frac{1}{1+\text{exp}\left(-\frac{V+34}{6.5}\right)}$	6	

	1	-	-	$\frac{1}{1+\text{exp}\left(-\frac{V+65}{6.6}\right)}$	$200+\frac{3200}{1+\text{exp}\left(-\left(V+63.6\right)/4\right)}$	

Pyr - KCa	2	$\frac{-0.00642\left(V_{s}+18\right)-0.1152}{-1+\text{exp}\left(-\left(V_{s}+18\right)/12\right)}$	$1.7\text{exp}\left(-\frac{V_{s}+152}{30}\right)$	$\frac{\alpha }{\alpha +\beta }$	$\text{max}\left(\frac{1}{\alpha +\beta },1.1\right)$	

		*V_s _*= *V *+ 40 log_10_(10^4^[*Ca*^++^]*_i_*)				

Int - Naf	3	$4.2\text{exp}\left(\frac{V+34.5}{11.57}\right)$	$4.2\text{exp}\left(-\frac{V+34.5}{27}\right)$	$\frac{\alpha }{\alpha +\beta }$	$\frac{1}{\alpha +\beta }$	

	1	$0.09\text{exp}\left(-\frac{V+45}{33}\right)$	$0.09\text{exp}\left(-\frac{V+45}{12.2}\right)$	$\frac{\alpha }{\alpha +\beta }$	$\frac{1}{\alpha +\beta }$	

Int - Kdr	4	$0.3\text{exp}\left(\frac{V+35}{10.67}\right)$	$0.3\text{exp}\left(-\frac{V+35}{42.68}\right)$	$\frac{\alpha }{\alpha +\beta }$	$\frac{1}{\alpha +\beta }$	

Gating functions [11] used for the membrane currents in the model neurons. The cell type and current are listed at the left, and the column head ins refer to Eq 5: gating exponent (*m*), gating variables (α and *β*), the equilibrium activation(inactivation) (*x*_∞_), and the activation(inactivation) time constant (*τ_x_*).

To model the response of excitatory synaptic inputs, we implemented an excitatory chemical synaptic input as in published models of AMPA and NMDA synapses [29]. When a presynaptic spike occurred at time *t_pre_*, a time dependent conductance was initiated that was based on a two state kinetic scheme [30] described by rise time constant (*τ_rise_*), and decay time constant (*τ_decay_*). The maximal inward depolarizing conductance (ḡ) was calibrated to generate physiological network behavior, and the reversal potential for these conductances, *V_glu _*= 0 mV [30]. The following equation describes the AMPA synaptic conductance (*g_glu_*) of both AMPA and NMDA receptors used in this model:

(6) $$g_{glu}\left(t-t_{pre}\right)=ḡ\left(e^{-\left(t-t_{pre}\right)/\tau_{decay}}-e^{-\left(t-t_{poe}\right)/\tau_{rise}}\right)$$

The synaptic current for each excitatory synaptic release was then calculated as, *I_glu _*= *g_glu_*(*V *− *V_glu_*). The Mg^2+ ^block for NMDAR is based on physiological concentrations of Mg^2+ ^by multiplying the current, *I_glu_*, by a voltage-dependent factor [30,31]. Inhibitory chemical synapses represent GABA-A receptors and are also implemented as a two state kinetic scheme [30] similar to the AMPA receptors. We use the GABA-A (chloride) reversal potential appropriate for the cell types.

In addition to the synaptic channels, AMPA and NMDA from glutamatergic neurons and GABA-A from interneurons, there are membrane ion-channels in all compartments. Every cellular compartment of both pyramidal cells and interneurons has a delayed rectifying K^+ ^(Kdr) channel, a fast Na^+ ^(Naf) channel and a leak channel. Pyramidal compartments also have a slowly inactivating K^+ ^(Ks) channel, a Ca^2+ ^mediated K^+^(KCa) channel, a persistent Na^+ ^(Nap) channel (only in some compartments) and a high-threshold L-type Ca^2+ ^(Hva) channel.

A stimulus is initiated by injecting a brief current at t = 2000 msec which starts the firing of the target pyramidal cells. Without further stimuli, this synchronized firing pattern goes on before it gets degraded by the background noise and the interference of the distractor neurons. This time span, called the working memory span, is usually in the range of 4-10 sec and corresponds to the time a certain pattern is held in working memory (for a review see [32]).

**Figure 3:**
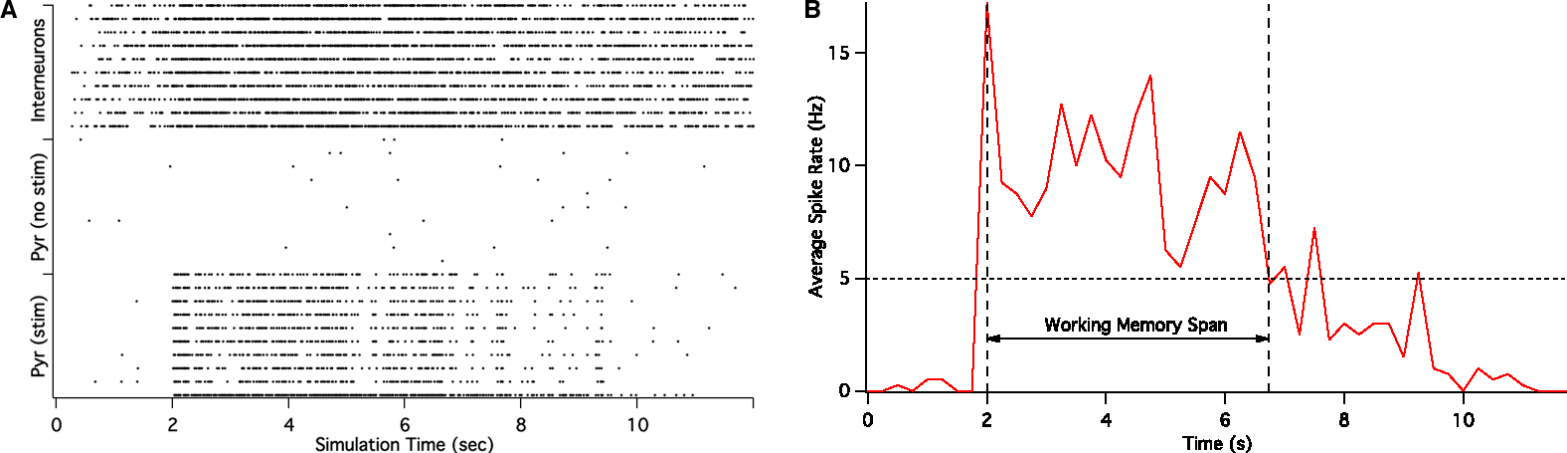
**Raster plot of the working memory model output**. A raster plot of the working memory model output (left panel) illustrates the concept of working memory span. Each line of asterisks is the activity of a single neuron with each asterisks indicating an action potential occurred at that time for the neuron. For simplicity we only show 10 excited pyramidal neurons, 10 unstimulated pyramidal cells and 10 GABA interneurons. At time 2 sec, a current (equivalent to a 'memory' stimulus) is injected into the attractor neurons (lowest ten lines) and these neurons then fire a sustained burst for a certain period until the stochastic noise starts to deteriorate the attractor pattern. The memory span is the time in seconds over which at least 80% of the attractor neurons fire within a similar time period (right panel).

This time span, called working memory span, is defined as the time a synchronous firing in the neurons that are stimulated is sustained without further stimulation. We first divide the time axis in bins of 200 msec and count the number of neurons firing in that time window and determine the time points where this number exceeds M/2, where M is the number of neurons stimulated at t = 2 sec (M = 40). The time difference between these two transition points is the memory span (Figure 3).

#### Implementation of receptor pharmacology

This subsection describes specifically the implementation of 5-HT and ACh neurotransmission physiology. Other neuromodulatory processes (dopaminergic (DA), noradrenergic (NE)) are implemented using similar approaches. In general, we assume a linear normalized relationship between receptor activation and biological effect on physiological responses such as$X_{Y}^{eff}=\left(X_{Y}^{A}-X_{Y}^{C}\right)/X_{Y}^{C}$, where $X_{Y}^{A}$ and $X_{Y}^{C}$ are the actual activation levels of receptor X subtype Y (for instance 5-HT_6_) after treatment (A) and the untreated (healthy) control levels (C).

5-HT_6 _receptor antagonism increases cortical ACh, DA and NE but not 5-HT with a maximal time-dependent effect of +200-250%; while the area under the curve is maximally increased tenfold [33]. 5-HT_6 _receptors are predominantly located in subcortical areas [34] and antagonism directly increases K^+ ^mediated ACh release in cortical and hippocampal slice [35].

With regard to subcortical DA activity; acute 5-HT_6 _receptor antagonism shifts DA firing in VTA from bursts to tonic firing with a maximum decrease of 35% in burst firing, while spontaneous activity of substantia nigra DA neurons increases by 40% after chronic 5-HT_6 _receptor antagonism [36].

We implemented the effect of 5-HT_6 _receptors in the cortical working memory model as an increase in free DA, NE and ACh that impacts activation levels of D_1_, D_2_, D_4_, M_1_, M_2_, *α*_7 _nicotinic ACh receptor (nACh-R), *α*_4_*β*_2 _nACh-R and α2A receptors. Let *P*_5*HT*6 _be a freely adjustable coupling parameter that will be fitted using clinical data. Given the actual, 5*HT*6*^A^*, and control, 5*HT*6*^C^*, activation levels of the 5-HT_6 _receptor, we calculate the effect on DA, NE and ACh receptors as,

(7) $$D_{x}^{*}=D_{x}\left(1+P_{5HT6}\frac{5HT6^{A}-5HT6^{C}}{5HT6^{C}}\right)$$

where x = 1,2, or 4 (dopaminergic receptors),

(8) $$M_{x}^{*}=M_{x}\left(1+P_{5HT6}\frac{5HT6^{A}-5HT6^{C}}{5HT6^{C}}\right)$$

where x = 1,2 (muscarinic receptors), and

(9) $$N_{x}^{*}=N_{x}\left(1+P_{5HT6}\frac{5HT6^{A}-5HT6^{C}}{5HT6^{C}}\right)$$

where x = *α*7 or *α*_4_*β*_2 _(nicotinic receptors).

Cholinergic physiology is implemented through the M_1 _receptor and both the *α*_7 _and the *α*_4_*β*_2 _nACh-R synapses, although pharmacology at the M_2 _receptor can play a role at this presynaptic autoreceptor. Their interactions are simulated using the cholinergic (muscarinic) receptor competition model. This interaction is important as many AD patients are on cholinomimetics such as acetylcholinesterase inhibitors.

Experimental data on pyramidal cells with ACh and specific M_2 _antagonists suggest that modulation of both M_1 _receptor and M_2 _receptor leads to a receptor activation-dependent average membrane potential change (Gulledge and Stuart, 2005, Gulledge et al., 2009), experimentally fitted with the following formula Δ*M(mV) *= −4 +6*A*_*M*1 _+2(1−*A*_*M*2_), where *A*_*M*1 _and *A*_*M*2 _are normalized M_1 _and M_2 _activation (both are bound between 0 and 1). This resting membrane potential change is caused by a change in K^+ ^channel conductance as *g'_Kdr _*= *g_Kdr_*(1 + Δ*M *· *P*_*M*1_), where *P*_*M*1 _is an adjustable parameter determined from clinical calibrations.

We implement the effect of *α*_7 _nACh-R modulation through presynaptic glutamate (Glu) release on Glu synapses that connect to pyramidal cells and interneurons through the following formula

(10) $$g_{x}^{*}=g_{x}\left(1+P_{M1}\frac{\alpha 7^{A}-\alpha 7^{C}}{\alpha 7^{C}}\right)$$

where *x *is either NMDA or AMPA.

Similarly *α*_4_*β*_2 _nACh-R regulates the GABA release at presynaptic afferent GABA neurons synapsing onto both interneurons and pyramidal cells with a coupling parameter $P_{\alpha_{4}\beta_{2}}$.

The parameters coupling the documented intracellular processes with these receptors are further calibrated using the correlation between the effect of therapeutic interventions in the network and their clinical working memory performance on the Alzheimer's Disease Assessment Scalecognitive subscale (ADAS-Cog) scale in Alzheimer's patients (as listed in Table 3).

**Table 3:** Clinical Alzheimer's Disease Assessment Scale-cognitive subscale (ADAS-Cog) used in the calibration.

Drug	Dose	Time (weeks)	ADAS-Cog Effect	Drug	Dose	Time (weeks)	ADAS-Cog Effect
Placebo	0	12	0.27	Galantamine	16	26	-1.10

Placebo	0	26	1.87	Galantamine	16	52	1.49

Placebo	0	52	5.07	Galantamine	24	12	-1.96

Placebo	0	78	7.10	Galantamine	24	26	-1.10

Donepezil	5	12	-1.77	Galantamine	24	52	1.49

Donepezil	5	26	-0.27	Rivastigmine	6	12	-0.66

Donepezil	5	52	2.87	Rivastigmine	6	26	0.70

Donepezil	10	12	-2.12	Rivastigmine	6	52	3.71

Donepezil	10	26	-0.64	Rivastigmine	12	12	-1.82

Donepezil	10	52	2.48	Rivastigmine	12	26	-0.77

Galantamine	8	12	-1.77	Rivastigmine	12	52	2.01

Galantamine	8	26	-0.95	SB-742457	5	26	-0.22

Galantamine	8	52	1.29	SB-742457	15	26	-0.66

Galantamine	16	12	-1.96	SB-742457	35	26	-1.12

Clinical ADAS-Cog changes for different drugs, doses and time points, used in the calibration of the AD network model. The values are derived from the population kinetic model based upon a meta-analysis and other reports on placebo and 5-HT_6 _antagonists.

There was no attempt to synchronize the activity of neuromodulatory pathways, such as dopamine and serotonin on the dynamics of the cortical network, because there are not enough data available. Many of these pathways fire tonically at low frequencies (1-5 Hz range), although short bursts might be present [37]. Since we are interested in somewhat longer-term properties (that is the capacity of sustained network activity over many seconds), we anticipate time-dependent changes in neuromodulatory pathways to have a limited impact. In addition, the pharmacodynamic half-life of drugs that change receptor activation levels is much longer than the tens of seconds the network activity is sustained. For instance the half-life of donepezil is well over 48 hr [38]. Therefore as a first approximation, the neuromodulatory effect is averaged over the full time range of the simulation.

#### Introducing pathology in the model

We implement AD pathology as a loss of cortical neurons [39] at a rate, *τ_N_*(%/week) and synapses from pyramidal neurons [40] with a rate *τ_S_*(%/week). Both excitatory-excitatory (e-e) and excitatory-inhibitory (e-i) synaptic connections are eliminated at the same rate, but because there is an additional pyramidal cell loss, e-e synapses tend to decrease faster. We constrain the distribution of neuronal loss so that the number of stimulated and unstimulated pyramidal cells must be the same.

Reduced cholinergic tone [41] is implemented as a free parameter on all cholinergic receptors (M_1 _and M_2 _muscarinic receptors, and α7 and *α*_4_*β*_2 _nicotinic receptors). In addition we introduced a placebo effect at week 12 using an increase in general cortical dopaminergic tone [42].

For the calibration of the Alzheimer disease network, we collected the publicly available data of either sex from clinical trials in mild-to-moderate AD patients using ADAS-Cog clinical scales (Table 3). The clinical effects of AChE-Is were studied in a population-kinetic model using a meta-analysis of all approved AChE-Is [43]. Using the parameters reported in [43], Table 2, we calculated the clinical responses of the placebo and AChE-Is.

In addition we also included reported clinical data on 5-HT_6 _antagonists [7] and placebo data at 78 weeks from tarenflurbil and tramiprosate trials [44-46]. Altogether we used 28 different clinical interventions over four time points (12, 26, 52 and 78 weeks), four drugs (donepezil, galantamine, rivastigmine and SB742457) with up to three doses. ApoE4 effects were introduced using specific synapse loss [47] and cholinergic deficit [48].

The calibration of the network was performed using design of experiment (DOE) statistical techniques, rather than one factor at a time (OFAT), because OFAT techniques are computationally intensive and are unable to detect interaction between parameters to be calibrated [49]. A good robust approach uses 2*n *simulations, where *n *is the number of free parameters, compared to 2*n *for a full OFAT design [50]. DOE techniques are computationally effective and provide a sound statistical approach to identify the driving parameters. Basically, this approach progresses in two parts. First a factorial approach identifies the major drivers of the optimization process and limits broadly the range of individual parameter settings, allowing fast optimization in a multi-dimensional parameter space. Second, a surface response analysis can be used to probe the (much smaller) parameter space in order to find the optimum.

### Calibration of serotonergic synapse

**Figure 4:**
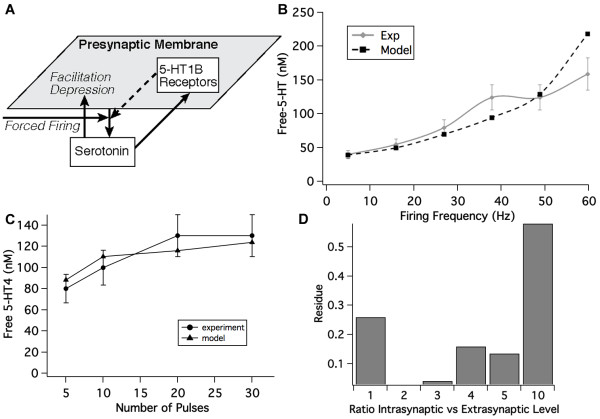
**Serotonin synapse modelling**. (**A**) Processes involved in measuring free serotonin levels by fast cyclic voltammetry. In this particular simulation only the change in free 5-HT, but no postsynaptic activation level is measured. The simulation setup forces high frequency firing on the presynaptic nerve endings and allows calibration of the different presynaptic parameters that regulate the coupling between presynaptic autoreceptor activation, firing history and subsequent neurotransmitter release. Some parameters, such as 5-HT release and half-life are constrained by biological limits. (B) Correspondence between model outcomes and experimentally reported values in the mouse substantia nigra under forced firing frequencies. The model parameters of the serotonergic synapse are adjusted to show a good correlation with the experimental data. This particular calibration is for the situation where the extrasynaptic concentration equals the intrasynaptic free level of 5-HT. (**C**) Correspondence between model outcomes and experimentally reported values [51] in the mouse dorsal raphe under forced firing frequencies where a specified number of pulses was given at 30 Hz. The model parameters of the serotonergic synapse are adjusted to show a good correlation with the experimental data. (**D**) Global difference between simulated and clinically measured data. Similar curve fittings to (**B**) are done for other situations (from 1 to 10 times the measured extrasynaptic levels). Because the *in vivo *serotonergic firing frequency is around 1 Hz, we took great care to fit the low end of the frequency datapoints.

Because 5-HT_6 _antagonism is extensively used in the calibration of the network, we first calibrate the human serotonergic synapse model, using a combination of *in vivo *experimental data on free 5-HT levels in preclinical animal models and human imaging data with specific radiotracers (Figure 4A).

We use fast cyclic voltammetry data in mouse slices [51] which have been shown to be rich in serotonergic innervation. Free 5-HT levels are measured after forced firing, providing experimental data for calibrating the presynaptic physiology. The 5-HT_1_*B *receptor is the most important autoreceptor for most projection serotonergic neurons, while 5-HT_1*A *_is the major autoreceptor regulating dorsal raphe firing [52]. Binding of 5-HT to the 5-HT_1_*B *receptor was described by affinity *K_i_*values of 23.4 nM for the antagonist tracer displacement and 4.2 nM for the agonist tracer while EC50 value was 8.5 nM [53]. We then adjusted the parameters of the presynaptic physiology until we obtained the best correlation between model outcomes and experimental values, and repeated this for different ratios of intra-synaptic over extra-synaptic free 5-HT.

We fitted the five parameters of the presynaptic physiology (relative sensitivity, facilitation weight, facilitation half-life, depression weight, depression half-life) to the 10 experimental results on the forced firing frequency (Figure 4B-C). A coarse parameter search was followed by the method of steepest descent to minimize the square value of the differences between experimental and model results. This resulted in the values of 7.75 for RelSens, 1.1 and 0.42 for facilitation and depression weight respectively and 90 and 120 msec for facilitation and depression half-life respectively.

An important issue is how much of the intrasynaptic 5-HT is detected by fast cyclic voltammetry probes. Modeling studies of the glutamate synapse [54] suggest that intrasynaptic levels can be between one and twenty times the measured extrasynaptic levels.

Because it is important to know the free 5-HT level in the human situation, the strategy is to systematically vary the ratio of extra- versus intrasynaptic free 5-HT, for each of these ratios, calibrate the 'mouse' 5-HT synapse, and then select the best ratio that has the highest correlation between the output of the synaptic model and actual clinical data on human serotonin imaging experiments. For this we simulated positron emission tomography (PET) radiotracer displacement human imaging studies using 5-HT receptor-specific radiotracers. We then selected the ratio between intra- and extrasynaptic free 5-HT that best fits the displacement of 5-HT_2*A *_receptor tracers in humans treated with certain serotonergic modulators with affinity for the 5-HT_2*A *_receptor, more specifically schizophrenia patients treated with antipsychotics.

We simulated the following clinical imaging experiments: treatment with 30 mg aripiprazole resulted in a 58% displacement of setoperone at the 5-HT_2*A *_receptor and over 90% displacement of raclopride at the D_2 _receptor [55]. Given the binding values for aripiprazole of 3.3 nM for D_2 _receptor, we can then calculate the functional free concentration of the drug at the striatal D_2 _receptor in that clinical situation, which allows us then to simulate the displacement of setoperone with aripiprazole (affinity 21.8 nM for 5-HT_2*A *_receptor), assuming the free concentration of aripiprazole is the same in the cortex as in the striatum.

For the other imaging studies at the 5-HT_2*A *_receptor, no displacement at the D_2 _receptor was described in the reported studies. We therefore determined the functional brain concentration of the antipsychotics for that particular clinical dose using the D_2 _receptor specific raclopride tracer imaging experiments reported for similar clinical doses in other patient groups in a primatized model of the striatal synapse [10]. Because raclopride displacement is measured functionally, it takes into account many confounding issues such as blood-brain barrier transport and free fraction and reflects the actual true concentration of the drug.

With the radiotracer altanserin, a 65% block was observed after 6 month treatment with 300 mg quetiapine [56]. The affinity of altanserin and quetiapine for 5-HT_2*A *_receptor was described with a *K_i_*value of 0.3 nM (Tan et al., 1999) and 264 nM, respectively. Using setoperone as a 5-HT_2*A *_receptor tracer, 80%, 90% and 10% displacement was observed with 600 mg chlorpromazine, 200 mg clozapine and 10 mg amisulpride, respectively [57], while a dose-range of 10 to 100 mg loxapine resulted in occupancy between 27 and 100% [58]. Similarly, 10 mg amoxapine displaced setoperone for over 90% [59].

For each of the conditions mentioned above, we then simulated the displacement of the tracers setoperone or altanserin by the appropriate functional brain concentration of the antipsychotic, given its known affinity for the human 5-HT_2*A *_receptor. Because the amount of tracer displacement results from a complex interaction between tracer, drug and serotonin, it will depend upon the absolute level of free 5-HT. We further assume that these values for the human 5-HT synapse can be extrapolated to normal healthy subjects or Alzheimer patients, as schizophrenia is mostly associated with a dopamine, but not a serotonergic dysfunction [60,61].

Using the functional concentrations of the antipsychotics derived from the raclopride displacement studies, we then simulated the displacement of 5-HT_2*A *_receptor tracer for the seven clinical cases for each of the ratios of intrasynaptic versus extrasynaptic 5-HT levels and compared the outcomes with the clinically reported data.

The calibration suggests that a ratio of intra- over extrasynaptic free 5-HT of 2 times gives the best correlation with the human imaging data (Figure 4C). We therefore used this value for all subsequent experiments with serotonin modulators.

### Target engagement of cholinergic and serotonergic drugs in AD clinical trials

#### Cholinergic drugs

Acetylcholinesterase inhibitors (AChE-I), such as donepezil, galantamine and rivastigmine have been tested extensively in the clinical AD trials. Imaging studies with acetylcholinesterase specific radiotracer 11C-PMP have reported inhibition levels of 40% for 10 mg donepezil and 12 mg rivastigmine and 35% for 16 mg galantamine [62-64] in the brain in Alzheimer's disease at clinically relevant doses.

Assuming a mass equation relationship, that is *K_i _*= *D*(1 − *l*)/*l*, where *D *is dose and *l *is inhibition level, we determined the clinical *K_i_*doses for AChE-I from the above reported inhibition levels as 15 mg for donepezil, 30 mg for galantamine and 18 mg for rivastigmine. Blocking the acetylcholinesterase enzyme to a level *l*, increases the half-life of free ACh in the synaptic cleft to *τ*_0_/(1 − *l*), where the basal half-life *τ*_0 _is 5 ms.

These changes in half-life of ACh were then applied in our cholinergic receptor competition model to determine the subsequent postsynaptic receptor M_1 _mACh-R, *α*_7 _nACh-R and *α*_4_*β*_2 _nACh-R activation, with the presynaptic cholinergic receptor being of the M_2 _muscarinic type for each of the doses of the AChE-I. We further assumed linear pharmacokinetics relative to the dose of the AChE-I.

#### Serotonergic drugs

The 5-HT_6 _antagonist SB742457 is currently in Phase II for Alzheimer's disease and clinical results as a stand-alone medication have been reported [6,7] for 5, 15 and 35 mg. Using target engagement studies [65], these doses have been shown to displace 84, 94 and 97% of the radiotracer GSK215083. We determined the functional concentration of SB742457 corresponding to these tracer displacement values and subsequently the resulting postsynaptic 5-HT_6_-R activation level for the 3 doses of SB742457 using the calibrated humanized 5-HT synapse. This resulted in postsynaptic 5-HT_6_-R activation levels of 40.6%, 12.9%, 5.1% and 3.1% for placebo, 5, 15 and 35 mg of SB742457 respectively.

## Results

We extended a biophysical model of cortical circuitry that was calibrated with primate electrophysiology data [11]. Although this model is very detailed and complete, it has been calibrated using only single-unit electrophysiology data in preclinical animal models. In addition, the absence of neuromodulatory drug targets and clinical calibration make the model less useful for supporting research and development programs. Our extensions of the model are to (1) implement the physiology of a number of neuromodulatory receptors based upon preclinical physiology, (2) increase the size of the network to 80 pyramidal cells and 40 inhibitory interneurons to accommodate higher resolution for the AD pathology, (3) reduce the relative fraction of inhibitory synapses according to recent neuroanatomical data, (4) implement the pathology of Alzheimer's disease based upon human pathology data and (5) calibrate the remaining biological coupling model parameters using the correlation between the effects of therapeutic interventions and genotypes in the model and the reported ADAS-Cog clinical effects. The model includes the physiology of the dopamine D_1_, D_2 _and D_4 _receptors, the serotonin 5-HT_1*A*_, 5-HT_2*A*_, 5-HT_3_, 5-HT_4 _and 5-HT_6 _receptors, the adrenergic α2A receptor and cholinergic M_1_, M_2 _mACh-R, and *α*_7 _and *α*_4_*β*_2 _nACh-R, whereas the pharmacology of 5-HT_6 _antagonism includes increases of glutamate and acetylcholine in the cortex.

### Calibration neuronal and synapse loss in the Alzheimer's disease model network

#### Prodromal AD stage

This section is devoted the calibration of synapse and neuronal loss occurring during amnestic MCI, a prodromal stage leading up to the clinical diagnosis of AD. This transition point is characterized by a precipitous drop in cognitive performance. We therefore determined the minimal loss of neurons and synapses where the performance of the network starts to break down.

**Figure 5:**
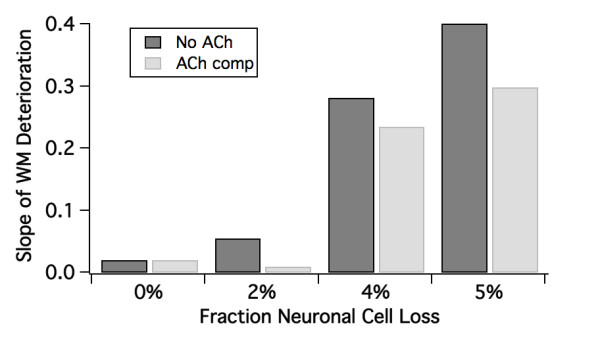
**Working memory loss**. Slope of working memory loss (defined as the decrease of working memory span per percent of deleted synapses) with increasing fractions of neuronal cell loss. It is clear that from 4% the decrease in performance accelerates substantially. As expected, having a greater cholinergic compensation tends to attenuate the decrease in working memory performance.

In a systematic parameter space of synapse and neuronal loss between 0 and 5% we determined that neuronal loss of 3-4% and beyond leads to substantial decrease in working memory performance. Be-cause of the finite nature of the network (80 pyramidal cells), real differences are seen at 2, 4 and 5% loss. We also introduced a 'cholinergic compensatory' mechanism which raised the levels of free ACh in the cortex by 10% and consequently increased activation of M_1_, M_2 _muscarinic receptors and of *α*_7 _and *α*_4_*β*_2 _nicotinic receptors. Based upon these simulations, we determine the threshold for AD to be at 5% neuronal cell loss (Figure 5).

#### Progression of Alzheimer pathology

Once we had determined the minimal deletion of synapses and neurons that corresponds to the transition between MCI and AD, we then proceeded to calibrate the remainder of the free parameters that describe the progression over time, the placebo effect and the coupling of the various pharmacological interventions.

**Figure 6:**
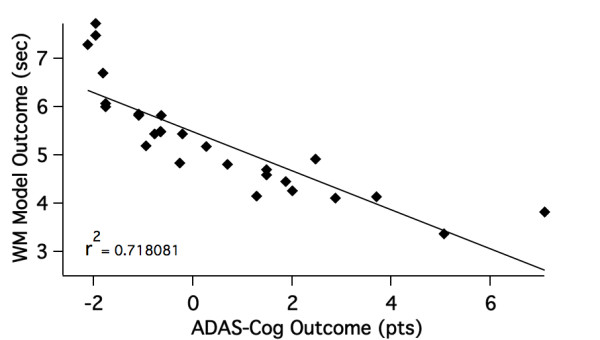
**Figure 6 Global correlation between Alzheimer's Disease Assessment Scale-cognitive subscale (ADAS-Cog) outcomes and model predictions**. Global linear correlation between reported ADAS-Cog outcomes from 28 different therapeutic interventions and their corresponding working memory effects in the computer model of Alzheimer's disease (*P <*0.0001). Because ADAS-Cog readouts monitor errors, positive values are associated with shorter working memory spans and worse performance.

We first applied a DOE approach to find the optimum in the 7-dimensional parameter space, using a 2*n *(14) runs matrix to achieve a first identification of the drivers for this optimization. Basically for each of the 14 different combinations of minimum and maximum values we calculated the correlation coefficient between model outcome for the 28 different clinical conditions and their reported clinical results and the corresponding slope. The Pareto plots suggested that the major drivers for the optimization of the correlation were %-synapse loss and %-neuronal cell loss. After three iterations and 17 further optimization steps we arrived at the correlation between model outcome and clinical ADAS-Cog scales in Figure 6.

Table 4 shows the optimal set of parameters as determined by the previous experiments. When using a linear fit, the correlation is *r*^2 ^= 0.73 with these parameters. When fitting with a second degree polynomial, the correlation increases to *r*^2 ^= 0.87 (data not shown).

**Table 4:** Free parameters

Name	Description	Biological Range	Optimal Value	Reference
Slope %Syn	Slope of fraction of synapses disappearing/week	Max 0.075%/week, on top of neuronal loss	0.004	[94]

Slope %Neuron	Slope of neurons eliminated/week	Max 0.5%/week, leads to 50% neuron loss in 100 weeks	0.025	[78]

ACh deficit	Size of the cholinergic NBasalis deficit	Range 5-50% loss	0.175	[41,95]

5-HT6 effect	Relation between 5-HT6 inhibition and free DA, ACH and NE increase	Maximum 0.20	0.025	[33]

DA increase in 12 wk placebo	DA surge from reward circuit that simulates placebo effect	Maximal 20% (tracer displacement in volunteers)	0.075	[42,96]

Rel *α*7 vs. *α*4*β*2 nACh-R effect	Relative effect of α7 over *α*4*β*2 nACh-R mediated effects	Depends upon dose and nature of enhancement Range 0.4-2.0	2	[97]

AChE-I effect on M1 receptor activation	AChE-I increases M1 mACh-R activation level to make pyramidal cells more excitable	Maximal change in membrane resting potential -8 mV (depolarizing)	0.075/8 mg Gal or equiv	[98]

List of 7 free parameters that were calibrated using the relation between clinical outcomes and working model outcomes. We report also the neurophysiological implementation and the biologically realistic boundaries, together with the value determined for the optimal correlation. With these 7 parameters on a database of 28 individual data points we achieve a correlation of *r*^2 ^= 0.73.

**Figure 7:**
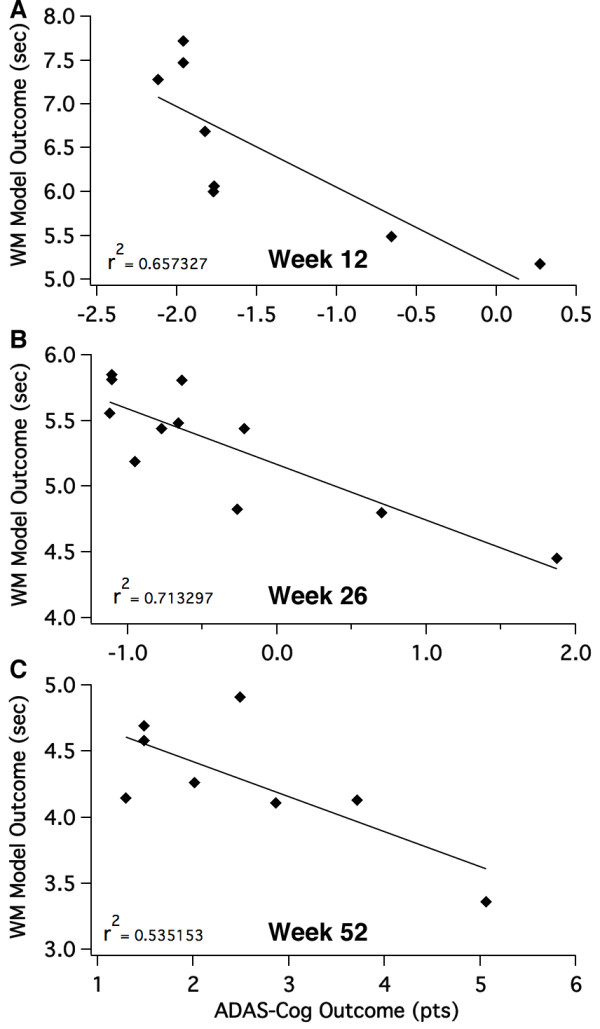
**Weekly correlation between Alzheimer's Disease Assessment Scale-cognitive subscale (ADAS-Cog) outcomes and model predictions**. (**A**) Correlation between changes in reported ADAS-Cog outcomes from 7 different therapeutic interventions at week 12 and their corresponding working memory effects in the computer model of Alzheimer's disease (*P *= 0.038). For the time points, the data show that even for individual time points there is a good correlation between clinical outcome and corresponding computer model outcome. (**B**) Correlation between reported ADAS-Cog outcomes from 12 different therapeutic interventions at week 26 and their corresponding working memory effects in the computer model of Alzheimer's disease (*P *= 0.042). (**C**) Correlation between reported ADAS-Cog outcomes from eight different therapeutic interventions at week 52 and their corresponding working memory effects in the computer model of Alzheimer's disease (*P *= 0.031).

When studying the correlation between clinical outcomes and model outcomes at different time points (Figure 7), the calibration results in (1) a correlation coefficient of *r*^2 ^= 0.58, a slope of -1.4 ADAS-Cog points/WM sec, and p = 0.02 for the 8 points at 12 weeks; (2) a correlation coefficient of *r*^2 ^= 0.47, a slope of -2.7 ADAS-Cog points/WM sec, and *P *= 0.013 for the 11 points at 26 weeks; (3) a correlation coefficient of *r*^2 ^= 0.52, a slope of -3.7 ADAS-Cog points/WM sec, and *P *= 0.035 for the 8 points at 52 weeks. This suggests that the model is able to capture the individual differences of each therapeutic intervention and the global correlation is not driven prominently by the differences between outcomes at 12 and 78 weeks. The data also suggest that the slope of ADAS-Cog points per working memory second increases from -1.45 at early time points (corresponding to mild AD) to -3.72 at later time points (corresponding to moderate AD).

Interestingly, the optimal parameter relating M_1 _receptor activation change to degree of AChE inhibition corresponds to the increase in postsynaptic M_1 _receptor due to the increased ACh half-life as determined. For 8 mg galantamine this resulted in 9.1% increase in M_1 _receptor activation. This suggests that the network automatically converges towards a biologically reasonable value for this particular free parameter.

#### Response surface analysis

In this subsection we studied the sensitivity of the calibration parameters, i.e. how do small changes in parameters affect the value for the slope between changes in working memory span (sec) and corresponding changes in ADAS-Cog points.

A sensitivity analysis was performed by keeping all but one parameter constant and testing the parameter region around the value identified in the full-factorial approach. We then determined how much the location of the optimum is dependent on the value of a particular parameter. Ideally one would not accept substantial changes in the correlation outcome with small changes in a specific parameter.

Studies of changing the ACh deficit coupling in the range 0.075 to 0.4 revealed only a 10% decrease in the correlation value around the optimum point (0.15). In addition the slope of working memory span change in seconds to ADAS-Cog clinical points change similarly, between -2 and -2.7 ADAS-Cog points/sec.

Similarly, the sensitivity of the slope outcome with regard to the 5-HT_6 _coupling effects showed even less sensitivity. Over a range from 0.005 to 0.04, around the optimal value of 0.025, the correlation value decreases less than 10%.

Changing the range of synapse loss slope (percent synapses lost per week pathology), between 0.028 and 0.043 resulted in a monotonic decrease in working memory, as expected, especially at the earlier stages (12 and 26 weeks). The value for the correlation varies within 14% and for the slope within 18%. Although somewhat greater than the values for ACh deficit and 5-HT_6 _effects, this suggests a limited sensitivity of the optimal parameter choice to the parameter of synapse loss.

As expected, neuronal loss pathology has the biggest effect on the network performance. This is the most sensitive parameter being fit. However, the value we chose (0.25%) has the highest correlation. For values between 0.2 and 0.5; the correlation ranged from a maximum of 0.73 to 0.39 and the slope of the correlation function ranged from -1.5 to -2.7.

### The effect of APOE genotype on the network performance in AD

The most important risk factor for AD is the ApoE4 genotype [66]. While the exact molecular sequence is currently unknown, many data suggest a decreased synapse density [47] and a lower cholinergic tone [48] to be associated with the ApoE4 genotype. We therefore implemented these effects in the network model for both the MCI and the AD case.

**Figure 8:**
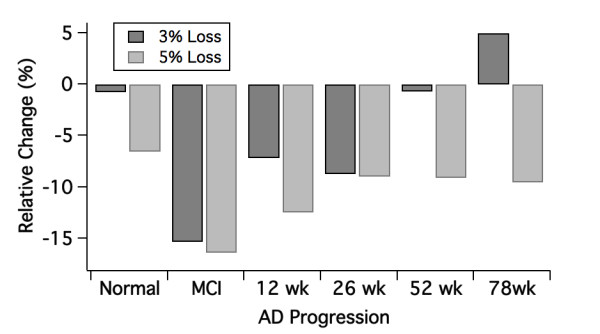
**Model validation with ApoE4 genotype in the Alzheimer's disease (AD) cortical network**. Effect of additional synapse loss and cholinergic tone decrease as part of the ApoE4 genotype in the AD cortical network on relative changes in network working memory span. We assume a 3% loss for 1 ApoE4 allele and a 5% loss for two ApoE4 alleles. The datasuggest that the biggest negative effect of synapse loss is observed in the early stages.

Simulation results in Figure 8 show that the effect of synapse loss is more pronounced in the earlier stages of the pathology than in the later stages. As expected, there is a dose-dependent decrease of working memory performance with increasing additional loss of synapses.

An additional 3 or 5% loss of synapses on top of the already lower synapse density as part of the AD pathology has the biggest effect at the early stages, that is, when the number of lost synapses is relatively lower. For instance going from a 3% synapse loss to a 6% synapse loss with 3% neurons lost (MCI case) has a much larger effect than going from 7 to 10% synapse loss with 23% neurons lost (the 52 week case). Using the calibration data, a 10-15% decrease in network performance at earlier AD stages corresponds to a difference of 1-1.5 points on the ADAS-Cog scale. This result is qualitatively in line with clinical data suggesting that APOE has its biggest effect on the age of onset for AD [67], that is, in the very early prodromal stages of AD but much less on the progression of the disease once diagnosed [68].

For the cholinergic compensation in the MCI state, we assumed a 10% increase in Ach levels and calculated the corresponding change in postsynaptic mAChR and nAChR. Such a value is in line with the compensatory differences in ChAT staining observed in the brain of MCI patients [69]. The cholinergic deficit in the AD pathology state was fixed at 30% decrease and was unchanged over the progression of the disease. On top of this deficit, APOE4/4 carriers had an additional Ach decrease that was calculated with the receptor competition model to satisfy the imaging studies of the M2 specific radiotracer [48]. Figure 8 shows the effect of the cholinergic compensation to attenuate the deficit slope: from 0.06 to 0.01 for a neuronal cell loss of 2%, from 0.28 to 0.23 at 4% and from 0.40 to 0.30 at 5% neuronal cell loss.

### The effect of memantine in different AD pathology conditions

Memantine is a weak NMDA antagonist that is approved for moderate to severe Alzheimer's disease [70]. Interestingly the drug has been shown to decrease cognitive performance in healthy volunteers [71], improve cognitive function in mild-to-moderate AD patients [72], but has the greatest effect in later moderate to severe AD stages [8,73], where it is approved. We explore here the glutamatergic component of memantine. Recent studies suggest that memantine under physiological concentrations of Mg^2+ ^inhibits the NMDA receptor NR2C/D subunits more than the NR2A/2B subunits [74]. Based upon the observation that the NR2C/2D subunits are preferentially located on inhibitory interneurons [75] in rats, we explored whether a greater inhibition of the NMDA receptor on interneurons would result in a differential effect of memantine at later stages of the AD pathology.

Data suggest that functional memantine concentration in the human brain is relatively small; together with the intrinsic pharmacology as a weak and uncompetitive inhibitor at the NMDA receptor [76], suggesting a small decrease in NMDA functionality.

**Figure 9:**
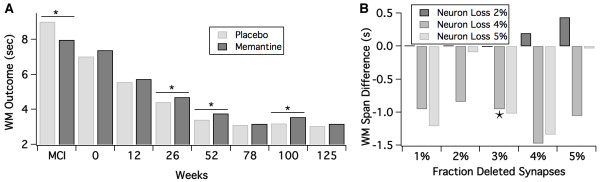
**Effect of memantine pharmacology**. (**A**) Effect of memantine pharmacology as represented by a 0.5% decrease in N-Methyl-D-aspartic acid (NMDA) conductance on excitatory-excitatory and 1% decrease of NMDA conductance on excitatory-inhibitory glutamate synapse on cortical AD network outcome. Memantine leads to a deterioration in the very early stage MCI (mild cognitive impairment) that further turns into a stronger positive effect as the pathology (i.e. loss of synapses and neurons) advances. (**B**) Sensitivity analysis of the difference between memantine and placebo effect for different values of amount of synapse and neuronal cell loss. The MCI state is defined as 3% synapse loss and 4% neuronal cell loss (indicated by ★ on the figure). A negative outcome corresponds to a decrease in performance after memantine. The effect of memantine is conserved over a range of synapse loss (1-5%) and neuronal cell loss (4-5%).

Figure 9A shows the effect of a decrease of 0.5% in the excitatory NMDA receptor and 1% in the inhibitory NMDA receptor on the network performance. These simulation results suggest that memantine decreases performance in normal individuals, but tends to improve the network performance better at later stages of the AD pathology, when more excitatory neurons are eliminated. In contrast, a hypothetical compound that blocks both NMDA subtypes to the same degree, leading to the same level of inhibition at both excitatory and inhibitory glutamatergic synapses improves the network performance at all pathology stages. This differential effect of memantine, especially the decrease at the earlier stages was only observed at relatively low values of memantine-induced NMDA conductance effects (that is,<2%).

The calibrations on the ADAS-Cog suggested a baseline of 5% synapse loss and 5% neuronal cell loss at time 0 of the clinical trial; therefore an MCI pathology is defined as a loss of 3% for synapses and a loss of 4% for neuronal cells. This leads automatically to the results observed in Figure 8 that suggest a worsening effect of this pharmacology in earlier stages of the pathology while providing a beneficial effect at later stages. We subsequently did a sensitivity analysis around the differential effect of memantine versus placebo in different pathological states of MCI (Figure 9B); suggesting that the decrease in working memory readout is relatively independent of the level of synapses deleted or the fraction of neurons deleted (4 or 5%). The lack of differentiation for a neuronal cell loss of 2% probably reflects the saturation of network readout.

## Discussion

This report describes the implementation and calibration of a computer-based mechanistic and biophysically realistic neuronal network model for working memory with Alzheimer's pathology. Although such network models have been developed in the past [11,77], they have not been made actionable for the modeling of drug effects and pharmaceutical research and development. A position paper [9] suggests that an upscaling of modeling from intracellular and molecular modeling to network and circuit modeling is essential for computational disease modeling. We believe that our combination of the receptor competition model, which describes the effect of drugs on the receptor activation level, with cortical network modeling of biophysically realistic neurons might be a first step in that direction.

A major difference to previous pharmacological research using modeling and simulation is the implementation of the proper target engagement by the drugs in the clinical situation. This is necessary in order to determine the receptor or target activity level at the appropriate clinical drug concentration. We first calibrated the receptor competition model using preclinical fast cyclic voltammetry data on serotonin in rodents [51]. As these experimental results likely reflect extrasynaptic neurotransmitter levels, we then constrained the relation between intra- and extrasynaptic values using PET displacement studies in humans using appropriate 5-HT and DA tracers. In order to get functional antipsychotic drug concentrations in the model, we simulated the tracer displacement experiment in human patients using this calibrated receptor competition model, which eliminates basically all issues about brain penetration. Finally we determined the effect of these drugs on the average activation level of the different receptor or target subtypes which then were entered in the network model.

Another major improvement to make this platform actionable for drug discovery and development is parameter calibration using correlation between model outcome and clinical results from retrospective data. Ideally all values for the coupling between neuromodulatory receptor activation and downstream effects on ion channel conductances should be derived from actual molecular biological measurements; however the only available data are from rodents or primates. Traditionally modeling studies calibrate the parameter set using the similarity with electrophysiological outputs from rodents and non-human primates. Our starting point was a cortical model [11] that was been calibrated using single-unit recordings in primates. As the human neurophysiology might be substantially different from the rodent or primate neurophysiology and to make it more translational, we used the correlation between model outcome and actual clinical test results on a cognitive test to determine the calibration parameters.

The results with the retrospective clinical data suggest that the calibration slope of ADAS-Cog points is about -2.8 points/sec of working memory and that this slope is steeper at longer time points (or more severe pathology), that is, a slope of -3.7 ADAS-Cog points/sec working memory at 52 weeks and a slope of -1.5 ADAS-Cog points/sec working memory at 12 weeks. It is unclear whether this corresponds to a 'cognitive' inflection point as has been suggested [78].

The progression of the neuronal cell loss in placebo conditions is about 0.35%/week, which corresponds to 18% per year or 5.2 years for a total decrease. This is in line with estimates for the time it takes for intraneuronal paired helical fillaments to become a ghost tangle: about 3.4 years in the hippocampus CA1 regions and 5.4 years in the subiculum [79].

In this model, we assumed that the major effect of the cholinomimetics was through their effect on acetylcholinesterase. Rivastigmine is the only drug that also blocks the enzyme butyrylcholinesterase (BuChE). However, the contribution of BuChE to the degradation of ACh in the cortex is relatively unknown. The major effect of rivastigmine, a combination of its activity against BUChE and the pseudo-irreversible cholinesterase inhibition, is the differential up-regulation of AChE and BuChE in the cerebral spinal fluid of patients after chronic treatment [80]. Rivastigmine is the only drug that reduces both activity and protein levels in the cerebral spinal fluid. Given the complex and undocumented nature of the relation between cerebral spinal fluid levels and actual brain activity levels of AChE and BuChE, we did not include the up- or down-regulation of the cholinesterases over time for this numerical model.

The cholinergic pathology is implemented as a loss of neuronal fibers with the assumption that the remaining cholinergic innervation function normally, that is, with the same presynaptic kinetics. This resulted only in a decrease of the amount of ACh released that had further impacted the activation levels of the postsynaptic muscarinic and nicotinic receptors.

We further assumed that the 5-HT dynamics were unchanged over the course of the disease pathology. There is indeed evidence that the noradrenerge system in the locus coeruleus [81], but not the dorsal raphe 5-HT is affected in AD pathology [82]. If new data become available, for instance using specific radiotracers, this could easily be introduced in the mechanistic disease platform.

An important test of any numerical model is the reproduction of clinical results not used for calibration. The observation that the model qualitatively reproduces both the observed clinical effects of APOE genotype and memantine likely increases the confidence in the model's predictions.

Selective synaptic loss affects the model outcome much more in earlier stages of the pathology in the network, likely because this is a proportional bigger decline in functional synapse loss than at later stages. Assuming that this synapse loss is a major hallmark of the ApoE4 genotype [47], this is qualitatively more in line with clinical data [67,68] that suggest that the APOE genotype has its biggest effect in determining the age of onset.

Similarly, the model qualitatively recapitulates the differential clinical beneficial effects of memantine, a weak NMDA antagonist, as a function of the pathology [8,71]. This is not a trivial result because reducing NMDA activity in preclinical models has been consistently associated with a decline in cognitive performance. Analyzing the model results suggests that a relatively greater inhibition by memantine of the NMDA receptor synapsing upon inhibitory interneurons by its virtue of its preferential block the NMDA receptor NR2C subunit can explain this apparent contradiction.

Interestingly, in our computational neuronal network model without AD pathology, the effect of blocking inhibitory NMDA receptor has always more impact than reducing NMDA receptor on excitatory synapses to the same degree, leading to an enhanced excitation or working memory span. This makes it even more difficult to detect a global inhibitory effect when blocking the inhibitory NMDA receptor more than the excitatory NMDA receptor as in the case of memantine. Only the combination of a specific pathology and the specific memantine differential effect on both types of NMDA receptor leads to the observed decreased working memory span. Therefore it is unclear if the absolute range of values for the memantine induced effects would translate into the clinical situation, because they might be related to the specifics of the computer model. Nevertheless, it is of interest that this model provides an explanation of the differential effect of memantine in diverse pathology states.

Memantine might have other pharmacological targets beyond the NMDA receptor [83] and it has been argued, based upon rodent animal studies, that the clinical effect of memantine is unlikely to be driven by its glutamatergic action [84]. When focusing on the glutamatergic component in this quantitative systems pharmacology model, the data indeed suggest that the clinical observation of differential effects in early stage versus more severe AD can only be reproduced when the effect is limited to a very small range of NMDA receptor inhibition. As this model has been extensively based upon primate electro-physiology data this could be a result of the consequences of differential inhibitory tone in primates versus rodents. It has indeed been shown that primate inhibitory tone is more pronounced than rodent inhibitory tone as a consequence of faster firing dynamics and higher interneuron density [85]. In short, this is an example where this type of quantitative systems pharmacology can help elucidate important clinical questions and translational divergences between rodent and humans.

A limitation of the model is the relatively low number of neurons (120) used in this simulation paradigm. Other approaches such as the Blue Brain project use massive parallel computing to simulate a neocortical column consisting of 10,000 3D digitizations of real neurons and that are populated with model ion channels constrained by the genetic makeup of over 200 different types of neurons [86]. The small number of neurons in the network might seem to be a concern; however recent studies in primates suggest that the behavior in a visual working memory paradigm can be described by the activity of only a few hundred cells [87]. In addition, the validation results suggest that a comparatively small network model can reveal drug mechanisms of action and guide drug development.

Another limitation is the fact that the model uses a very simplified representation of the pyramidal neuron and the inhibitory interneuron. While the small number of neuronal compartments might speed up the calculation, it is by far not a complete representation of a human neuronal cell. In addition, the model uses only one type of interneuron, the fast-spiking basket interneuron, while it is known that the human cortex has many different types [88]. For other types of physiological readouts, such as the simulations of brain oscillations, there may be a need for multiple different types of interneurons. However, even such a simplified network is able to qualitatively represent the emergent property of a stable activity pattern that mimics a working memory property, as evidenced by the similarity of the model outcome with electrophysiology readings in primates performing such a task [11]. This is not unlike the observation that many different parameter sets might lead to identical emergent properties [89].

Although working memory decline is certainly present in the disease and seems to be sensitive to cholinomimetic treatment, only a small part of the ADAS-Cog is really dependent on working memory [90]. Indeed, a small study with 5mg donepezil in both elderly controls and mild AD patients documented a significant improvement in Groton maze learning [91], a task involving spatial working memory and error monitoring. AD patients seem to perform better in complex working memory tasks of high value, but not low-value items [92]. Another way to interpret the results from this network model is to consider the model as measuring the stability of a memory representation, irrespective of the nature of the cognitive task.

## Conclusions

The results presented here demonstrate that quantitative systems pharmacology approach can be complementary to traditional animal models to predict the efficacy of pharmacological treatments for patient subtypes. This type of systems level computational modeling has the potential to assess potential off-target effects, the consequences of pharmacologically active human metabolites, the effect of comedications, and the impact of well described genotypes. Our particular implementation of quantitative systems pharmacology can generally aid drug research and development in the central nervous system as it uses human-specific properties of candidate drugs, such as more realistic drug exposure, specific pharmacology against the human receptors, the pharmacological effect of unique human metabolites and functional genotypes unique to the human situation with an effect on cognitive outcome [93]. It recapitulates part of the neurophysiology and neuropathology of Alzheimer's diseases, and although incomplete in physiological details, can help in assessing the effect of human-specific properties of clinical candidates and improve the clinical success rate.

## Abbreviations

5-HT: 5-hydroxytryptamine; ACh: Acetylcholine AChE-I: Acetylcholinesterase inhibitor AD: Alzheimer's disease; ADAS-Cog: Alzheimer's Disease Assessment Scale-cognitive subscale; AMPA: -amino-3-hydroxy-5-methyl-4-isoxazolepropionic acid ApoE4: Apolipoprotein E 4; DA: Dopamine; DOE: Design of experiment; GABA: gamma-Aminobutyric acid; mACh-R: Muscarinic acetylcholine receptor MCI: Mild cognitive impairment; NE: Norepinephrine; NMDA: N-Methyl-D-aspartic acid; NR2C: NMDA receptor subunit type 2C; NR2D: NMDA receptor subunit type 2C; OFAT: one factor at a time; PET: Positron emission tomography; VTA: Ventral tegmental area.

## Competing interests

The authors are employees of *In Silico Biosciences, Inc*, and the company has financed this manuscript and holds a patent on the computational platform that was used to arrive at the results of this article.

## Authors' contributions

All authors contributed equally to the simulation design and execution. HG conceived of the study, and drafted the manuscript. All authors contributed equally in the editing of the manuscript. All authors read and approved the final manuscript.
